# Flipover outperforms dropout in deep learning

**DOI:** 10.1186/s42492-024-00153-y

**Published:** 2024-02-22

**Authors:** Yuxuan Liang, Chuang Niu, Pingkun Yan, Ge Wang

**Affiliations:** Biomedical Imaging Center, Center for Biotechnology and Interdisciplinary Studies, Department of Biomedical Engineering, Rensselaer Polytechnic Institute, Troy, NY 12180 USA

**Keywords:** Model robustness, Regularization, Flipover, Dropout, Adversarial defense

## Abstract

Flipover, an enhanced dropout technique, is introduced to improve the robustness of artificial neural networks. In contrast to dropout, which involves randomly removing certain neurons and their connections, flipover randomly selects neurons and reverts their outputs using a negative multiplier during training. This approach offers stronger regularization than conventional dropout, refining model performance by (1) mitigating overfitting, matching or even exceeding the efficacy of dropout; (2) amplifying robustness to noise; and (3) enhancing resilience against adversarial attacks. Extensive experiments across various neural networks affirm the effectiveness of flipover in deep learning.

## Introduction

In recent years, deep learning has demonstrated significant success across diverse fields, spanning computer vision, natural language processing, medical imaging and drug design. Properly designed and trained artificial neural networks can adeptly model intricate patterns and nuances derived from extensive data. However, as model complexity grows, challenges emerge, notably ensuring the model robustness, particularly under noisy or adversarial conditions.

In deep learning models, robustness is defined by the ability to produce consistent and reliable outputs amidst shifts and perturbations in the input data. The variations alter the distribution of the input data from that of the training data [1], with the most prevalent case being the shift from the training dataset to the testing dataset. Model lacking robustness, exemplified by over-fitting, may excel on training data but often fails on unseen data, resulting in sub-optimal real-world performance [2]. Several methods have been proposed to address over-fitting and other issues, such as refs. [3, 4]. An established measure for evaluating model robustness is its ability to handle noisy input data. For instance, an image classifier should identify an input image even with added noise [5]. Furthermore, robustness indicates resilience against adversarial attacks. With the growing use of deep learning models in critical applications, their susceptibility to adversarial attacks has been investigated. Adversarial attacks use crafted input data in such a way that they mislead the model to make incorrect predictions, while being almost indistinguishable from the original data [6]. The adversarial defense has emerged as a prominent topic in deep learning with multiple strategies [7–9]; however, these defenses are often computationally expensive.

Among techniques developed to improve model robustness, dropout, introduced by Hinton et al. [10], stands out as an effective yet simple method. Dropout introduces randomness into the model by sporadically setting a fraction of input units to zero during training. This strategic noise incorporation deters neuron co-adaptation, improving generalization and preventing over-fitting. Initially applied on fully connected models, dropout has since been expanded to various deep neural networks, including convolutional neural networks (CNNs) and transformers. Modifications to the original dropout method include DropConnect [11], DropBlock [12], MaxDropout [13], and spectral dropout [14].

Deep neural networks are increasingly applied to complex tasks, necessitating stronger regularization strategies. While dropout is effective in preventing overfitting, it offers a limited enhancement of noise and adversarial robustness. Specialized adversarial defense algorithms are effective, while often incurring computational overhead [15]. Thus, algorithms that bolster the comprehensive robustness without incurring high computational costs are required. Existing research demonstrates that multiplying the model weights using Gaussian noise in the form of $N(1,\sigma )$, which is positive, can outperform standard dropout [16]. Here, this study proposes an upgraded version of dropout, named ‘flipover’, which can improve the model’s robustness from new angles. Unlike dropout, flipover does not merely zero out certain features. Instead, it employs a bolder approach, multiplying a selection of the original features using a negative factor, for instance, -1. This approach does not merely remove features; it introduces opposite features as perturbations, challenging the model to learn from an altered feature representation. The preliminary work indicates that flipover can (1) prevent over-fitting as effectively as standard dropout, (2) improve noise robustness, which is not the primary focus of dropout, (3) facilitate adversarial defense because flipover generates adversarial attacks efficiently. While not designed initially for adversarial defense, Wang et al. [17] reported that applying dropout during testing enhances model performance under adversarial attacks. However, incorporating a large dropout factor during testing can substantially diminish the model’s effectiveness on the original dataset. Conversely, our method, when employed with appropriate techniques, minimally impacts the original model’s performance.

**Fig. 1:**
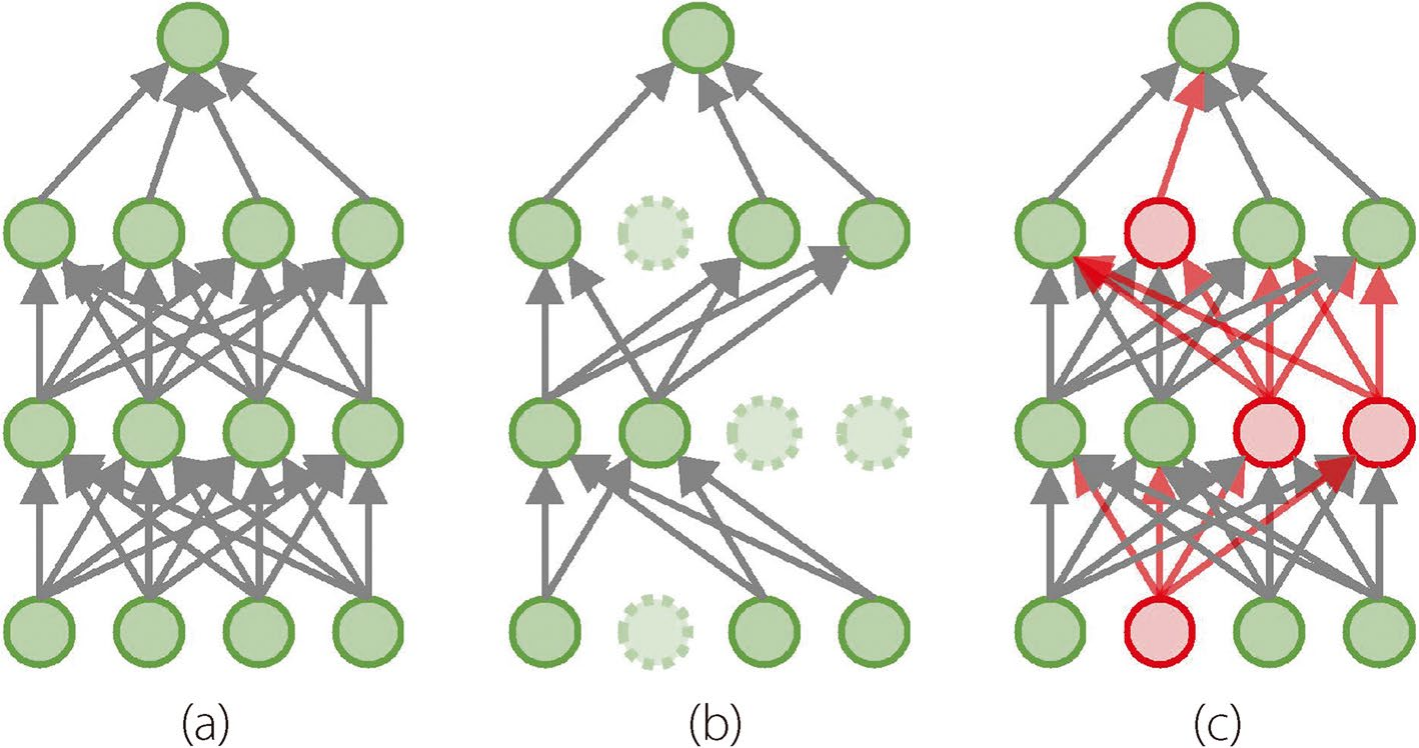
Illustration of flipover operations. (**a**) A standard feedforward network; (**b**) The network with dropouts; (**c**) The network with flipovers

## Methods

### Algorithm description

Figure 1 illustrates a standard fully-connected network, and its modifications with either dropouts or flipovers. Dropout randomly removes some neurons such that they do not work during the feed-forward or back-propagation processes. Conversely, flipover modifies a certain proportion of neurons by a negative multiplier in the hope that their negative effects help robustify the network. This section presents the flipover formulation, following the process of dropout illustrating flipover as a stronger regularization strategy. The subsequent section experimentally establishes the advantages of flipover over dropout in several significant cases.

First, dropout technique is formulated using standard fully-connected network with *L* hidden layers. Let $\varvec{y}^{(l)}$ denote the output of the $l^{th}$ layer ($l \in \{1, \ldots , L\}$ and $\varvec{y}^{(0)}$ be the inputs). The feed forward operation can then be computed as

1 $$\begin{aligned} y_i^{(l+1)} = f\left( \varvec{w}_i^{(l+1)} \varvec{y}^{(l)} +b_i^{(l+1)}\right) \end{aligned}$$

where *f* is the activation function and $\varvec{w}^{(l)}$ and $\varvec{b}^{(l)}$ are the weights and biases of the $l^{th}$ layer. With dropout, Eq.  (1) becomes

2 $$\begin{aligned} y_i^{(l+1)} = f\left( \varvec{w}_i^{(l+1)} \varvec{\hat{y}}^{(l)} +b_i^{(l+1)}\right) \end{aligned}$$

where $\varvec{\hat{y}}^{(l)} = \varvec{r}^{(l)} * \varvec{y}^{(l)}$ and $\varvec{r}^{(l)}$ is a vector of independent Bernoulli random variables each of which has probability *p* of being 1, i.e., $r_j^{(l)} \sim$ Bernoulli(*p*) [16]. Similarly, the forward operation of the flipover method is the similar to the dropout operation except for $\varvec{\hat{y}}^{(l)} = \left(-\varvec{\frac{1}{\alpha }}\right)^{\varvec{r}^{(l)}} * \varvec{y}^{(l)}$, where $\alpha$ is a factor to control the amplitude of the flipped variables. Hence, the elements of $\varvec{\hat{y}}^{(l)}$ have a probability of $(1-p)$ remaining in their original and *p* to be flipped.

In deep learning, cross-entropy loss and the least square (LS) loss are two commonly used loss functions. In the following two subsections, the study employs these two losses to prove that flipover is a regularization mechanism.

### Derivation for cross-entropy loss

First, cross-entropy loss is considered:

3 $$\begin{aligned} \mathcal {L} = -\sum \limits _{i} t_i \mathrm{{log}} \left( \hat{y}_i^{(L+1)}\right) \end{aligned}$$

where $t_i$ denotes the ground truth for the $i^{th}$ output. If the activation function is Sigmoid function, the gradient of the standard network’s loss $\mathcal {L}_N$ concerning $\varvec{w}_i^{(l+1)}$ can be computed as follows:

4 $$\begin{aligned} \frac{\partial \mathcal {L}_N}{\partial \varvec{w}_i^{(l+1)}} = \left( t_i-\hat{y}_i^{(L+1)}\right) \varvec{\hat{y}}^{(L)} \end{aligned}$$

After applying dropout, the gradient of the dropout network’s loss $\mathcal {L}_D$ becomes:

5 $$\begin{aligned} \frac{\partial \mathcal {L}_D}{\partial \varvec{w}_i^{(l+1)}} = \left( t_i-\hat{y}_i^{(L+1)}\right) \varvec{r}^{(L)}\odot \varvec{\hat{y}}^{(L)} \end{aligned}$$

where $\odot$ represents the inner product operation. Therefore, the effect of dropout is equivalent to applying mask $\varvec{r}^{(L)}$ to the gradient of the standard network. Given this dropout mask, the gradients during back propagation are scaled. This helps to prevent the weights from receiving large gradient updates, making them over-reliant on specific patterns or features in the training data. This has a similar effect as weight decay (such as L2 regularization) where the magnitudes of the weight updates are constrained, although the mechanism is different.

Similarly, the gradient involving flipover is

6 $$\begin{aligned} \frac{\partial \mathcal {L}_F}{\partial \varvec{w}_i^{(l+1)}} = \left( t_i-\hat{y}_i^{(L+1)}\right) \left( -\varvec{\frac{1}{\alpha }}\right) ^{\varvec{r}^{(L)}}\odot \varvec{\hat{y}}^{(L)} \end{aligned}$$

which is equivalent to applying a mask $\varvec{r}^{(L)}$ that prevents large gradient updates and adds perturbations to the direction of the gradient. It has been proved that gradient noise can be regarded as a smoothing factor, contributing to global convergence [18]. Compared to random noise, the study flipped the gradient components the opposite direction, which had stronger and more targeted effects.

### Derivation for LS loss

With simplifications, the LS loss function can precisely express the regularization term introduced by filpover in its exact form. The losses for the normal network $\mathcal {L}_N$ and dropout network $\mathcal {L}_D$ can be written as

7 $$\begin{aligned} \mathcal {L}_N = \frac{1}{2}\left(t - \sum \limits _{i=1}^n w_i'I_i\right)^2 \end{aligned}$$

8 $$\begin{aligned} \mathcal {L}_D = \frac{1}{2}\left(t - \sum \limits _{i=1}^n \delta _i w_iI_i\right)^2 \end{aligned}$$

where $I_i$ denotes the inputs to a certain layer, and $\delta _i \sim$ Bernoulli(*p*). $\delta$ is equal to 1 with probability p, and 0 otherwise. In this calculation, only consider a linear model without activation functions is considered. The gradient of the dropout network can be calculated as:

9 $$\begin{aligned} \frac{\partial \mathcal {L}_D}{\partial w_i} = -t\delta _i I_i + w_i\delta _i^2I_i^2 + \sum \limits _{j=1, j\ne i}^n w_j\delta _i\delta _j I_i I_j \end{aligned}$$

For simplicity, assume $w' = pw$. It turns to

10 $$\begin{aligned} \mathcal {L}_N = \frac{1}{2}\left( t - \sum \limits _{i=1}^n pw_iI_i\right) ^2 \end{aligned}$$

11 $$\begin{aligned} \frac{\partial \mathcal {L}_N}{\partial w_i} = -t\delta _ip_i I_i + w_ip_i^2I_i^2 + \sum \limits _{j=1, j\ne i}^n w_jp_ip_j I_i I_j \end{aligned}$$

The expectation of the gradient of the dropout network can be calculated as

12 $$\begin{aligned} E\left( \frac{\partial \mathcal {L}_D}{\partial w_i}\right){} & {} = -tp_i I_i + w_ip\delta _i^2I_i^2 + w_iVar(\delta _i)I_i^2 + \sum \limits _{j=1, j\ne i}^n w_jp_ip_j I_i I_j \nonumber \\{} & {} =\frac{\partial \mathcal {L}_N}{\partial w_i} + w_ip_i(1-p_i)I_i^2 \end{aligned}$$

Therefore, dropout can be treated as a regularization term for the original loss function. Following this, the loss of the flipover network can be expressed as

13 $$\begin{aligned} \mathcal {L}_F = \frac{1}{2}\left( t - \sum \limits _{i=1}^n (-1)^\delta _i w_iI_i\right) ^2 \end{aligned}$$

Here, $\alpha$ is set to 1 for convenient computation. Hence, the gradient is

14 $$\begin{aligned} \frac{\partial \mathcal {L}_F}{\partial w_i} = -t(-1)^{\delta _i} I_i + w_i(-1)^{2\delta _i}I_i^2 + \sum \limits _{j=1, j\ne i}^n w_j(-1)^{\delta _i+\delta _j} I_i I_j \end{aligned}$$

Let $$w'=(1-2p)w$$, then

15 $$\begin{aligned} E\left( \frac{\partial \mathcal {L}_F}{\partial w_i}\right){} & {} = -t(1-2p_i) I_i + w_iI_i^2 + \sum \limits _{j=1, j\ne i}^n w_jI_i I_j \nonumber \\{} & {} =\frac{\partial \mathcal {L}_N}{\partial w_i} + 2w_ip_i(1-p_i)I_i^2 \end{aligned}$$

Therefore, flipover can be treated as a stronger regularization strategy than dropout. When $\alpha$ is set to zero, flipover reduces to dropout. Overall, two hyper parameters exist: the flipover rate, which indicates the proportion of neurons that will be flipped, and the flipping amplitude, a negative number.

##  Results

### Experimental settings

**Dataset and network structure.** The proposed method was applied on two different neural network models, which yield promising results, demonstrating the effectiveness of the flipover concept across networks of varying scales. The first model is a small CNN consisting of four convolutional layers and two fully connected layers, and has been used as a standard model in many previous work [19–21]. The parameter settings proposed by Wang et al. [17] is used, and the model was trained on the Modified National Institute of Standards and Technology (MNIST) dataset [21]. The second model was ResNet18 [4], which was trained using CIFAR10 dataset [22].

**Implementation details.** For the small CNN network, a simple flipover between the two fully-connected layers is applied, with $\alpha$ set to 1. For ResNet18, since the network is deeper, adding flipover to a single layer is insufficient. In the PyTorch official documentation, ResNet18 is divided into four blocks, each containing two basic blocks, which consists of convolutional layers, batch normalization layers and down-sampling layers. Flipover was applied between the two basic blocks of the fourth block. Further, the original single fully-connected layer was replaced with two layers and flipover was applied between them. In this case, $\alpha$ was set to 0.5. For a fair comparison, dropout was applied at the same positions as flipover for all networks. First the small CNN was trained to demonstrate the effect of flipover on preventing over-fitting. Subsequently, both networks were trained and random noise was added on the test set to demonstrate the improvement in noise robustness. Finally, adversarial attack were performed on both networks and the accuracy under attack was compared among models without regularization and with either dropout or flipover.

### Results

**Fig. 2:**
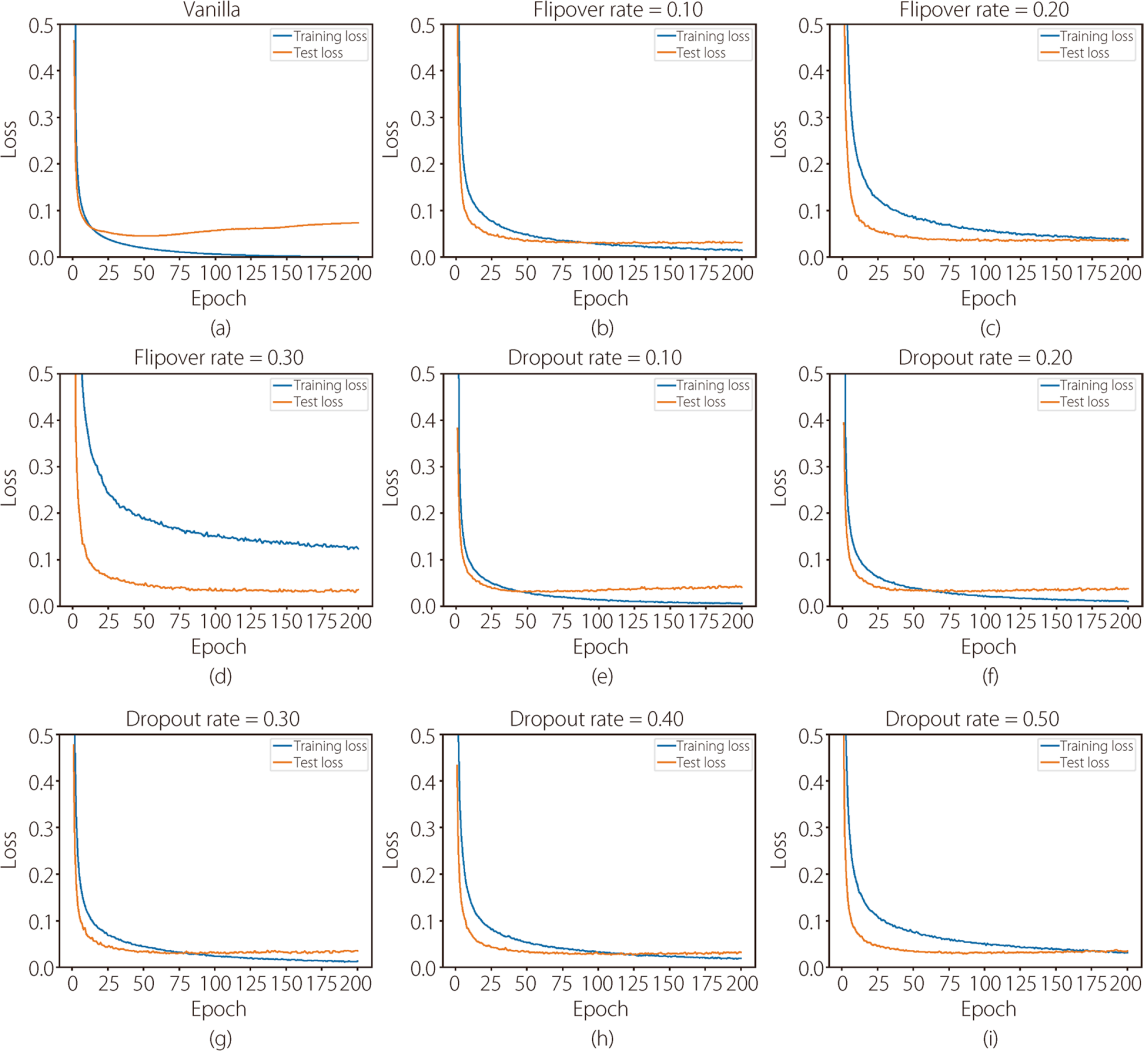
Training and test losses concerning different regularization strategies. (**a)** No regularization; (**b**)-(**d**) Flipover at different rates; (**e**)-(**i**) Dropout at different rates

**Overfitting prevention.** It was found that flipover was effective in preventing overfitting, as evidenced by plotting the training and test losses when training the small CNN on the MNIST dataset, as shown in Fig. 2. In the absence of any regularization, a clear pattern of overfitting emerged: the training loss consistently declined converging to zero, whereas the test loss initially decreased and then stopped at a significant level. When applying flipover, the test loss was effectively controlled, and its efficacy was directly proportional to the flipover proportion utilized. A flipover proportion of 0.2 outperformed the counterpart employing a dropout rate of 0.5. Essentially, the incorporation of flipover serves as a robust measure to counteract overfitting, enhancing the model’s generalizability and ensuring consistent performance across diverse datasets. However, when the flipover rate is high, the training loss converged to a relatively large value, which may cause a performance drop. Table 1 shows the test accuracy (ACC) for different regularization methods, where the Dropout/Flipover rate represents the probability that a neuron is dropped or flipped. With a flipover rate of 0.2, the model achieved the highest accuracy among all the settings.

**Table 1:** Test accuracy of the small CNN model on MNIST dataset

Method	Dropout/Flipover rate	ACC (%)
Vanilla	-	98.84
Dropout	0.1	98.94
	0.2	99.05
	0.3	99.07
	0.4	99.01
	0.5	99.00
Flipover	0.1	99.08
	0.2	**99.18**
	0.3	99.00

**Fig. 3:**
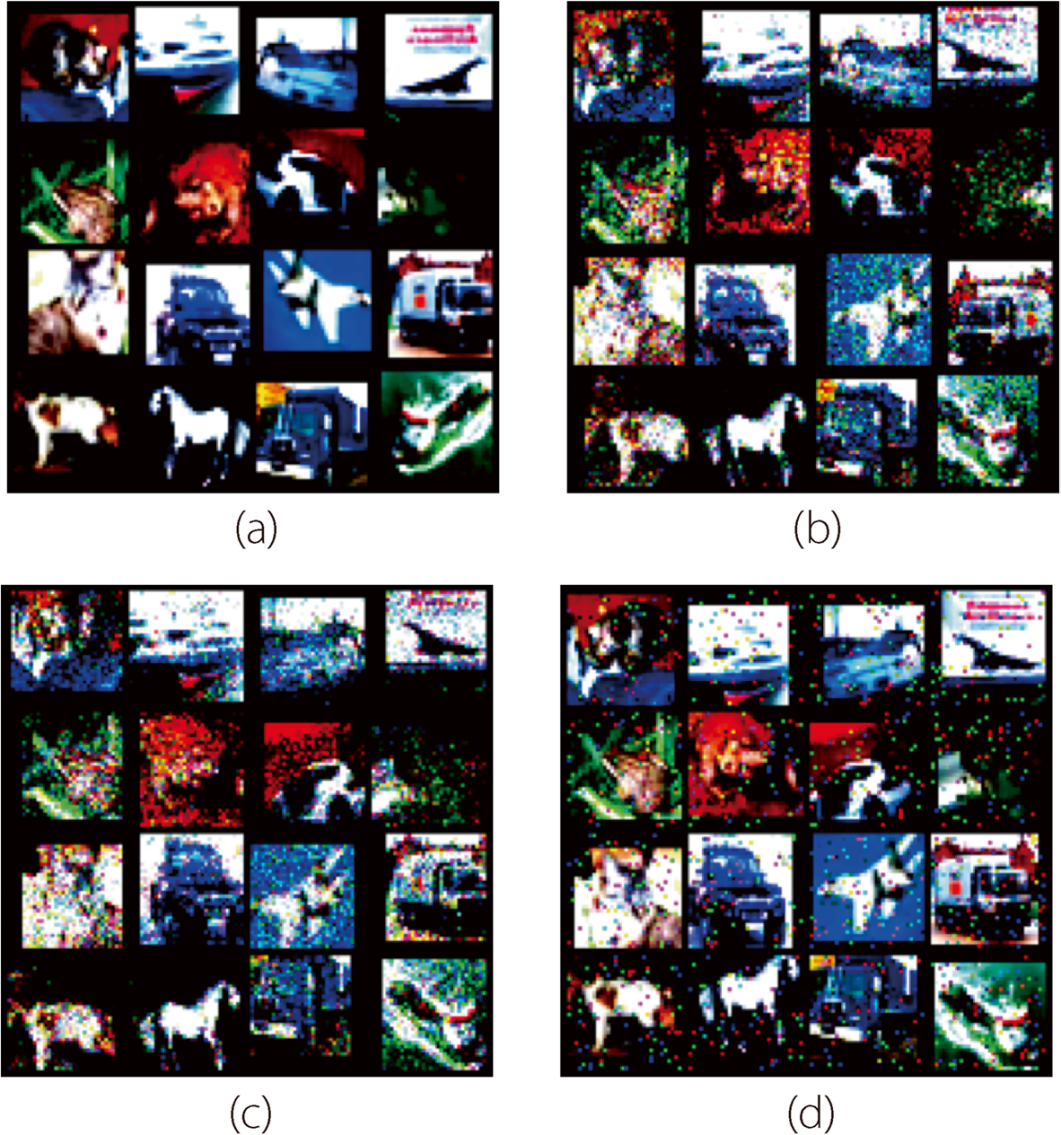
Examples of test data before and after adding Gaussian noise for the CIFAR10 dataset. (**a**) The original data without noise; (**b**) The data with Gaussian noise; (**c**) The data with Poisson noise; (**d**) The data with salt-and-pepper noise

**Noise suppression.** Flipover was applied to the small CNN and ResNet18 models to evaluate its effect on noise suppression. The models were trained on the original datasets and tested on noisy datasets generated by adding different types of noise to the original test sets. Three common types of noise in images were applied: Gaussian, Poisson, and salt-and-pepper. For the MNIST dataset, because Poisson noise did not significantly change the digital images, only Gaussian noise and salt-and-pepper noise were applied. The standard deviation of the Gaussian noise was set to 1.0, and the salt-and-pepper noise ratio was set to 0.4. For the CIFAR10 dataset, all three types of noise were applied. The standard deviation of the Gaussian noise was set to 0.1, the salt-and-pepper noise ratio was 0.05, and the scaling factor of the Poisson noise was 50 [23]. Figure 3 shows examples of the noisy data on the CIFAR10 dataset. Table 2 summarizes the experimental results. The models with flipover significantly outperformed the original models without regularization or with dropout. The results confirm the efficacy of flipover in enhancing the model’s resilience to noise, underscoring its potential as a tool for enahncing model reliability under noisy conditions.

**Table 2:** Noise suppression using the flipover technique

Model	Method	ACC Org (%)	ACC Gaussian (%)	ACC Poisson (%)	ACC salt (%)
Small CNN [17]	Vanilla	98.53	58.31	/	50.49
	Dropout	**98.60**	61.50	/	61.50
	Flipover	98.00	**68.71**	/	**69.62**
ResNet18 [4]	Vanilla	**93.63**	41.27	46.92	46.71
	Dropout	93.31	41.60	43.03	48.00
	Flipover	92.37	**46.50**	**49.85**	**53.49**

Note: ACC Org stands for the accuracy on the original test set; ACC Gaussian stands for the accuracy on dataset with Gaussian noise; ACC Poisson stands for the accuracy on dataset with Poisson noise; ACC salt stands for the accuracy on dataset with salt-and-pepper noise

**Fig. 4:**
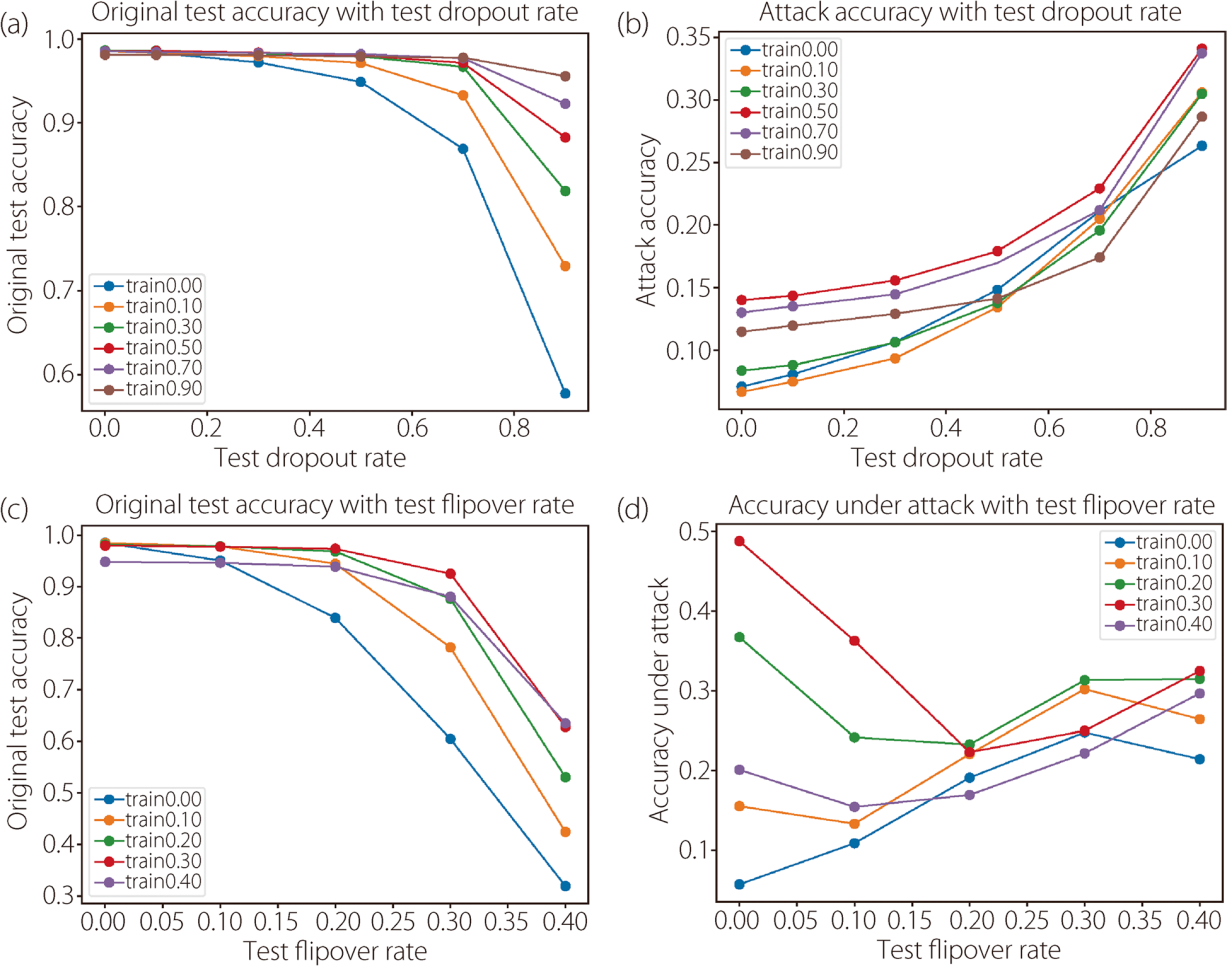
Adversarial defense capabilities of different methods. (**a**) Original test accuracies with different training and test dropout rates; (**b**) Test accuracies under attack with different training and test dropout rates; (**c**) Original test accuracies with different training and test flipover rates; (**d**) Test accuracies under attack with different training and test flipover rates

**Adversarial defense.** The efficacy of our method for adversarial defense is further assessed. The fast gradient sign method (FGSM) was applied [2] to attack the CNN and ResNet18 networks, and the flipover and dropout were respectively used for defense. A constant attack power of $\epsilon =0.25$ was maintained throughout the testing phase. In ref. [2], the authors separately set the training and test dropout rates from 0 to 0.9 to find the best combination of (training rate, test rate). This setting was followed with the flipover rate ranging from 0 to 0.4. Figure 4 shows the accuracy of the small CNN model under adversarial attacks with different combinations of training and test parameters. Table 3 lists the best results for flipover and dropout on both networks. On both the small CNN and ResNet18 networks, flipover achieve a much higher performance under attack than dropout. Furthermore, the flipover method has several advantages. Unlike dropout, which requires a high dropout rate at the test time for defense, flipover can achieve a decent defense effect when applied only during training. On ResNet18, dropout had almost no use under attack, while flipover still greatly improved the defense ability. Generally, the optimal combination of the flipover parameters is (0.3, 0.0), and with that settings, the original model accuracy was rather close to the vanilla models. By contrast, with the best combination of dropout parameters (0.7, 0.9), there was a substantial decrease in the original accuracy. Collectively, these findings underscore flipover’s preeminence in boosting adversarial robustness and enhancing the defense ability of a network without a concomitant compromise in model accuracy associated with dropouts.

**Table 3:** Adversarial robustness measures using no regularization, dropout, and flipover respectively

Model	Method	ACC Org (%)	ACC Att (%)	Para comb
Small CNN [17]	Vanilla	98.53	5.50	/
	Dropout	92.43	34.98	(0.7, 0.9)
	Flipover	98.00	**49.79**	(0.3, 0.0)
ResNet18 [4]	Vanilla	**93.63**	23.00	/
	Dropout	93.42	23.14	(0.0, 0.3)
	Flipover	92.37	**40.99**	(0.3, 0.0)

Note: ACC Org stands for the accuracy on the original test set; ACC Att for the accuracy under adversarial attack; Para Comb for the optimal combination of training and test rates

## Discussion

**Positions for flipover and parameter settings.** Because flipover is a stronger regularization strategy than dropout, the positions for implementing flipover and its parameters need to be carefully determined for optimal model performance, especially for deep neural networks. For small networks, flipover can be simply applied before the last few fully-connected layers, whereas for large networks more flipover operations can be added to ensure effectiveness. In general, flipover should be applied to the deeper layers of the network to avoid initial information loss. There are two parameters of the flipover operation: flipover rate and flip amplitude. Both affect the strength of the regularization. According to the experiments, for the selected network architectures, with the flipover rate of around 0.3 the model can improve robustness while not harming its accuracy. However, the flip amplitude can vary significantly in different models, and experientially, larger models generally prefer smaller amplitudes.

**Table 4:** Adversarial robustness measures for the transformer architecture using no regularization, dropout, and various flipover techniques respectively

Method	Dropout rate	Flipover rate	$\alpha$	ACC Org (%)	ACC Att (%)
Vanilla	-	-	-	81.63	9.50
Dropout	0.1	-	-	**82.88**	9.50
Replace all	-	0.1	0.5	80.45	**11.06**
Replace all	-	0.2	0.5	73.88	10.24
Replace all	-	0.1	0.7	78.95	7.74
Replace all	-	0.1	0.2	80.54	8.63
Replace gradual	-	0.1–0.3	0.5	73.88	10.24
Single	-	0.3	0.5	82.26	10.24

Note: Replace all means replacing all dropout layers with flipover; Replace gradual means replacing all dropout layers with flipover and gradually increasing the flipover rate for deeper layers; Single means adding a single flipover before the final head

**Effectiveness on transformer architecture.** Recently, the application of transformer-based networks has become widespread, surpassing that of traditional deep neural networks for various tasks including computer vision, natural language processing, and medical imaging. Recognizing the effectiveness of dropout in these networks, the potential benefits of implementing flipover is explored. A small vision transformer (ViT) model [24]is selected, which contains six transformer blocks with embedding sizes 512 and four heads. The network was trained on the CIFAR10 dataset and subjected it to FGSM attacks. Since ViT originally contained dropout layers, the initial attempt involved the straightforward replacement of the dropout layers with the flipover counterparts within the transformer blocks. However, this approach did not yield satisfactory results. Different strategies for incorporating flipover were explored and the findings compiled in Table 4. Although there was a notable improvement in the model’s accuracy under attack, the enhancement was not as significant as that observed for small CNN and ResNet architectures. Hypothetically, the moderate improvement can be attributed to the sub-optimal current integration of flipover in the transformer. The foundational elements of transformers, namely the attention mechanism [25] and embedding process [26], could be pivotal in this context. A more targeted approach, involving the introduction of perturbations within these core components could magnify the efficacy of flipover in defending against adversarial attacks.

**Combination with other regularization strategies.** It was found that flipover could be easily combined with other regularization methods. By adding a batch normalization layer to the small CNN model, the accuracy under attack reaches 67% coupled with flipover. As an ablation study, applying batch normalization only obtained results similar to what the vanilla model got (about 20%). This demonstrates the compatibility of flipover with other regularization methods.

**Limitations.** The proposed flipover method showed promising results in the experiments, surpassing the performance of the same models using standard dropout, yet the new method has its limitations. First, the decision of which layers to apply ‘flipover’ to requires deliberation. While larger networks generally benefit from more flipped layers, identifying the optimal strategy requires an empirical adjustment, which can be time-consuming. The flipover parameters, including the rate and amplitude, were manually determined. These fixed settings may not be optimal for other training scenarios. Further efforts are needed for optimal performance and generalizability. Notably, flipover, being a more intense form of regularization than dropout, can significantly impact the model performance if its strength is too strong. Therefore, caution must be exercised when setting a high flipover rate, as it may adversely affect the model performance. Finally, the use of flipover in large models is another interesting topic. For example, to regularize/stabilize a transformer architecture, the study hypothesizes that the attention mechanism should be the best target to perturb. This is currently being worked as a follow-up project.

## Conclusions

This study extended dropout to bolster model robustness on multiple fronts. The proposed approach not only serves as a more effective regularization technique than conventional dropout, mitigating overfitting, but also introduces adversarial perturbations to gradients, enhancing resilience against adversarial attacks. This makes the proposed method particularly suitable for challenging scenarios, including those involving noisy data, pronounced domain shifts, or direct adversarial confrontations. Optimal results requires judicious selection of positions in the network and network parameters for the flipover to maximize the benefits without compromising overall performance. Future research should focus on developing parameter selection strategies and tailoring this approach to transformer-based architectures.

## Data Availability

The public datasets used in this paper are open-access. The project code will be released upon acceptance.

## Abbreviations

CNN: Convolutional neural network
LS: Least square
ACC: Accuracy
FGSM: Fast gradient sign method
ViT: Vision transformer
MNIST: Modified National Institute of Standards and Technology

## References

[1] Fawzi A, Moosavi-Dezfooli SM, Frossard P (2017) The robustness of deep networks: A geometrical perspective. IEEE Signal Process Mag 34(6):50-62. 10.1109/MSP.2017.2740965

[2] Goodfellow IJ, Shlens J, Szegedy C (2015) Explaining and harnessing adversarial examples. arXiv preprint arXiv: 1412.6572

[3] Ying X (2019) An overview of overfitting and its solutions. J Phys: Conf Ser 1168(2):022022. 10.1088/1742-6596/1168/2/022022

[4] He KM, Zhang XY, Ren SQ, Sun J (2016) Deep residual learning for image recognition. In: Proceedings of 2016 IEEE conference on computer vision and pattern recognition, IEEE, Las Vegas, 27-30 June 2016. 10.1109/CVPR.2016.90

[5] Seltzer ML, Yu D, Wang YQ (2013) An investigation of deep neural networks for noise robust speech recognition. In: Proceedings of 2013 IEEE international conference on acoustics, speech and signal processing, IEEE, Vancouver, 26-31 May 2013. 10.1109/ICASSP.2013.6639100

[6] Zhou S, Liu C, Ye DY, Zhu TQ, Zhou WL, Yu PS (2023) Adversarial attacks and defenses in deep learning: from a perspective of cybersecurity. ACM Comput Surv 55(8):163. 10.1145/3547330

[7] Zantedeschi V, Nicolae MI, Rawat A (2017) Efficient defenses against adversarial attacks. In: Proceedings of the 10th ACM workshop on artificial intelligence and security, ACM, Dallas, 3 November 2017. 10.1145/3128572.3140449

[8] Benz P, Zhang CN, Karjauv A, Kweon IS (2021) Universal adversarial training with class-wise perturbations. In: Proceedings of 2021 IEEE international conference on multimedia and expo, IEEE, Shenzhen, 5-9 July 2021. 10.1109/ICME51207.2021.9428419

[9] Maini P, Wong E, Kolter JZ (2020) Adversarial robustness against the union of multiple perturbation models. In: Proceedings of the 37th international conference on machine learning, JMLR.org, 13-18 July 2020

[10] Hinton GE, Srivastava N, Krizhevsky A, Sutskever I, Salakhutdinov RR (2012) Improving neural networks by preventing co-adaptation of feature detectors. arXiv preprint arXiv: 1207.0580

[11] Wan L, Zeiler M, Zhang SX, LeCun Y, Fergus R (2013) Regularization of neural networks using dropconnect. In: Proceedings of the 30th international conference on machine learning, JMLR.org, Atlanta, 16-21 June 2013

[12] Ghiasi G, Lin TY, Le QV (2018) DropBlock: a regularization method for convolutional networks. In: Proceedings of the 32nd international conference on neural information processing systems, Curran Associates Inc., Montréal, 3-8 December 2018

[13] do Santos CFG, Colombo D, Roder M, Papa JP (2021) MaxDropout: deep neural network regularization based on maximum output values. In: Proceedings of the 25th international conference on pattern recognition, IEEE, Milan, 10-15 January 2021. 10.1109/ICPR48806.2021.9412733

[14] Poernomo A, Kang DK (2018) Biased Dropout and Crossmap Dropout: learning towards effective dropout regularization in convolutional neural network. Neural Netw 104:60-67. 10.1016/j.neunet.2018.03.016 10.1016/j.neunet.2018.03.016 29715684

[15] Akhtar N, Mian A, Kardan N, Shah M (2021) Advances in adversarial attacks and defenses in computer vision: a survey. IEEE Access 9:155161-155196. 10.1109/ACCESS.2021.3127960

[16] Srivastava N, Hinton G, Krizhevsky A, Sutskever I, Salakhutdinov R (2014) Dropout: a simple way to prevent neural networks from overfitting. J Mach Learn Res 15(1):1929-1958

[17] Wang SY, Wang X, Zhao P, Wen WJ, Kaeli D, Chin P, Lin X (2018) Defensive dropout for hardening deep neural networks under adversarial attacks. In: Proceedings of 2018 IEEE/ACM international conference on computer-aided design, IEEE, San Diego, 5-8 November 2018. 10.1145/3240765.3264699

[18] Qin XL, Xu X, Luo XP (2022) Global convergence of noisy gradient descent. In: Proceedings of 2022 IEEE international conference on systems, man, and cybernetics, IEEE, Prague, 9-12 October 2022. 10.1109/SMC53654.2022.9945497

[19] Papernot N, McDaniel P, Wu X, Jha S, Swami A (2016) Distillation as a defense to adversarial perturbations against deep neural networks. In: Proceedings of 2016 IEEE symposium on security and privacy, IEEE, San Jose, 22-26 May 2016. 10.1109/SP.2016.41

[20] Carlini N, Wagner D (2017) Towards evaluating the robustness of neural networks. In: Proceedings of 2017 IEEE symposium on security and privacy, IEEE, San Jose, 22-26 May 2017. 10.1109/SP.2017.49

[21] Lecun Y, Bottou L, Bengio Y, Haffner P (1998) Gradient-based learning applied to document recognition. Proc IEEE 86(11):2278-2324. 10.1109/5.726791

[22] Krizhevsky A (2009) Learning multiple layers of features from tiny images. Dissertation, University of Toronto

[23] Dytso A, Vincent Poor H (2020) Estimation in Poisson noise: properties of the conditional mean estimator. IEEE Trans Inf Theory 66(7):4304-4323. 10.1109/TIT.2020.2979978

[24] Dosovitskiy A, Beyer L, Kolesnikov A, Weissenborn D, Zhai XH, Unterthiner T et al (2021) An image is worth 16x16 words: transformers for image recognition at scale. In: Proceedings of the 9th international conference on learning representations, OpenReview.net, 3-7 May 2021

[25] Vaswani A, Shazeer N, Parmar N, Uszkoreit J, Jones L, Gomez AN et al (2017) Attention is all you need. In: Proceedings of the 31st international conference on neural information processing systems, Curran Associates Inc., Long Beach, 4-9 December 2017

[26] Mikolov T, Sutskever I, Chen K, Corrado G, Dean J (2013) Distributed representations of words and phrases and their compositionality. In: Proceedings of the 26th international conference on neural information processing systems, Curran Associates Inc., Lake Tahoe, 5-10 December 2013

